# Impact of Taper Design on Cleaning Efficacy, Stress Generation, and Irrigant Performance: A Combined Experimental, Finite Element Analysis, and Computational Fluid Dynamics Assessment

**DOI:** 10.3390/dj14020108

**Published:** 2026-02-13

**Authors:** Celia Vinuesa Maqueda, Natalia Navarrete, Ana Ramírez-Muñoz, Ana Martín-Díaz, César de Gregorio, José Aranguren, Giulia Malvicini, Simone Grandini, Gaya C. S. Vieira, Alejandro R. Pérez

**Affiliations:** 1 Department of Endodontics, Rey Juan Carlos University, 28032 Madrid, Spain; cvinuesam@hotmail.com (C.V.M.); anaamartindiaz@gmail.com (A.M.-D.);; 2 Faculty of Biomedical and Health Sciences, Universidad Europea de Madrid, 28670 Madrid, Spain; 3 Unit of Endodontics and Restorative Dentistry, Department of Medical Biotechnologies, University of Siena, 53100 Siena, Italysimone.grandini@unisi.it (S.G.); 4 Surpreendente Research Group, 4400-239 Vila Nova de Gaia, Portugal

**Keywords:** nickel–titanium instruments, cleaning efficacy, taper design, finite element analysis, computational fluid dynamics

## Abstract

**Background/Objectives:** This study aimed to compare the cleaning efficacy, biomechanical stress distribution under simulated occlusal loading after instrumentation, and irrigant dynamics of three NiTi rotary systems, namely ProTaper Gold, TruNatomy, and SlimShaper, using a combined experimental, finite element analysis (FEA), and computational fluid dynamics (CFD) approach. **Methods:** Transparent 3D replicas of mandibular mesial roots filled with a gel-like pulp tissue were instrumented with the three systems (*n* = 13 per group). Standardized irrigation was performed with 4% NaOCl delivered through IrriFlex^®^ needles positioned 2 mm from the working length. Cleaning effectiveness was assessed through digital image analysis, FEA simulation of occlusal loading, and CFD evaluation of irrigation flow, wall shear stress, and dynamic pressure. **Results:** All systems left residual tissue, with no statistically significant differences in cleaning efficacy among them (*p* > 0.05). Descriptively, ProTaper Gold showed the lowest mean residual tissue (0.15 ± 0.25%), followed by SlimShaper (2.50 ± 3.81%) and TruNatomy (4.20 ± 5.12%). CFD revealed that ProTaper Gold generated the highest irrigant velocities and wall shear stresses, while SlimShaper showed the highest dynamic pressure. FEA indicated that ProTaper Gold produced the highest stress concentrations, especially in the pericervical dentin, whereas TruNatomy and SlimShaper preserved more dentin. **Conclusions:** Cleaning efficacy was comparable across systems. CFD/FEA from representative models illustrated patterns of irrigant dynamics and dentin preservation without supporting system superiority.

## 1. Introduction

The long-term success of root canal treatment depends mainly on the effective elimination of bacteria, pulp remnants, and necrotic debris from the root canal system [1,2]. However, complete debridement remains a clinical challenge due to its high anatomical complexity [3]. Features such as isthmuses, lateral canals, and apical ramifications often harbor tissue and biofilm even after chemomechanical preparation [2]. Studies have shown that a considerable percentage of canal walls remain unprepared after root canal instrumentation [2,3], especially in mesial roots of mandibular molars, which compromises the effectiveness of disinfection [2].

Furthermore, bacterial persistence has been detected even after thorough chemical and mechanical preparation [2], especially in apical and irregular areas [2,3]. While sodium hypochlorite (NaOCl) remains the irrigant of choice [4], its efficacy depends on delivery technique and canal accessibility [5]. Although conventional irrigation with a syringe and needle is widely used, it has considerable limitations, particularly in reaching and effectively irrigating the apical third [2]. This is partly due to inadequate penetration of the needle when conservative shaping techniques are used and air entrapment or vapor lock formation in closed-end systems [6].

The design of NiTi instruments, especially their taper, plays a crucial role in facilitating irrigant flow and improving penetration [7]. Larger taper preparations allow the irrigation needles to reach closer to the working length (WL), enhancing apical cleaning [7]. However, this is at the expense of increased dentin removal, particularly in the pericervical region, a structurally critical area [8]. Excessive shaping in this region has been associated with an increased risk of vertical root fractures [9], emphasizing the need to balance canal accessibility and dentin conservation [8]. Recent biomechanical studies have highlighted how structural design and material removal patterns significantly influence stress distribution and deformation in dental tissues, ultimately affecting long-term mechanical stability [10,11]. Within this context, understanding how different endodontic shaping strategies influence post-treatment biomechanical behavior has become increasingly relevant to support dentin preservation-oriented therapeutic approaches [10,11,12].

For this reason, newer shaping systems with minimal or variable taper, which aim to preserve root dentin and reduce the risk of fracture, have been introduced [8]. However, such conservative preparations may limit the depth of needle insertion, which may compromise the efficacy of irrigation [6]. This limitation can be partially overcome by using a flexible, non-metallic irrigation needle such as Irriflex (Produits Dentaires SA, Vevey, Switzerland), which adapts better to canal curvature and allows deeper insertion into narrow preparations [13], improving the delivery of irrigation fluid and contact with the walls even in minimally shaped canals [13].

In order to evaluate the biomechanical effects of shaping, finite element analysis (FEA) is a useful tool to simulate occlusal loading and map stress distributions in endodontically treated teeth, allowing scenario-based comparisons of how post-shaping geometries might influence structural response [14]. Separately, computational fluid dynamics (CFD) provides detail on irrigant behavior inside shaped canals, including velocity fields, pressure, and wall shear stress, key variables for understanding syringe irrigation performance within complex anatomies [15].

Despite the growing interest in understanding the biomechanical and hydrodynamic behavior of instrumented root canals, most studies have evaluated either FEA or CFD in isolation [7,14,15], with limited connection to clinical cleaning results.

In the present study, each modality was applied for a distinct purpose and interpreted within its own scope: (i) an experimental in vitro assessment quantified the capacity to remove/dissolve a gel surrogate under a standardized syringe irrigation protocol (primary outcome); (ii) FEA was used to simulate how shaping with each system could affect stress distribution under occlusal loads (structural context); and (iii) CFD was used to visualize irrigant flow and wall interaction in the shaped canals with a standardized needle position (fluid dynamics context).

The aim of this study was to compare, in vitro, (a) cleaning performance (residual gel) as the primary experimental endpoint, and to provide (b) biomechanical (FEA) and (c) hydrodynamic (CFD) descriptions associated with three NiTi rotary systems of different tapers. The null hypothesis was that there would be no significant difference between the three NiTi systems regarding residual tissue, irrigation dynamics, or stress distribution after instrumentation.

## 2. Materials and Methods

### 2.1. Sample Size Calculation

The present study was conducted according to the Preferred Reporting Items for Laboratory Studies (PRILE) guidelines [16]. The Ethics Committee of the University of Siena, (Siena, Italy) approved the protocol (N.7/2021). The sample size was calculated a priori using G*Power (version 3.1) based on a one-way ANOVA model with an effect size of 1.2, a significance level (α) of 0.05, and a power (β) of 0.80. The calculation resulted in a minimum of 12 samples per group; however, 13 specimens per group were included to increase robustness and compensate for potential variability.

### 2.2. Tooth Model and 3D Replica Preparation

A natural mandibular molar with a well-defined mesial root was scanned in a Phoenix V|tome|x S240 (GE, Boston, MA, USA) using the following parameters: isotropic voxel size 20 µm, 155 kV, 190 µA, 360° rotation, 0.2 mm Al filter; reconstruction with Datos|x 3D (ring artifact correction 5, beam hardening 50%, smoothing 8; ~1300 images/tooth). The reconstructed root model showed two mesial canals with a Vertucci type IV configuration and an apical isthmus connecting the canals. The overall curvature of the root was approximately 20°, and the mesial root length was standardized to 21 mm in both mesial canals across all replicas.

The initial canal diameter was standardized to ISO [17] size 10. The master model was standardized to ISO #10 at D0 using the micro-CT-derived lumen. Minor deviations in visual appearance can occur due to segmentation smoothing and photopolymerization overcure in SLA printing; nonetheless, functional confirmation with a #10 K-file at WL was performed in all replicas before shaping to enforce the intended starting size. A conservative access cavity was prepared and standardized using the Meshmixer software (version 3.5; Autodesk Inc., San Rafael, CA, USA) to provide a uniform entrance to the canal.

The 3D replicas were printed using a high-precision Photon Mono M5s stereolithography printer (Anycubic Technology Co., Shenzhen, China) with a UV-resistant transparent resin (Anycubic Technology Co., Shenzhen, China) to enable visualization of the internal anatomy. All replicas were generated from the same master mold derived from a single micro-CT scan to ensure identical internal geometry and minimize volumetric variability between specimens, and were fabricated under standardized printing parameters as follows: XY pixel size = 10 µm; layer thickness = 20 µm; normal exposure = 1.6 s; off time exposure = 0.5 s; bottom exposure = 20 s × 3 layers; lift distance = 5 mm; lift/retract speeds = 8/8 mm/s. Printed parts were washed in IPA for 3 min, air-dried, and post-cured for 10 min at 405 nm, 60 °C.

Printing orientation and supports were standardized to avoid stair-stepping in the apical third, and all replicas were produced from the same STL to guarantee identical internal geometry. These settings were established in pilot prints to consistently reproduce both mesial canals and to achieve faithful resolution of the apical isthmus region, ensuring lumen continuity in the apical zone.

The internal canal spaces were filled with a red, protein-based hydrogel to simulate pulp tissue. We used a slightly modified formulation, relative to commonly used surrogates, to ensure readily NaOCl-dissolvable behavior. Specifically, agar and xanthan were omitted, and the gelatin content was increased, allowing the gel to be oxidatively degraded/solubilized by 4% sodium hypochlorite. Specifically, 6 g of gelatin (Merck, Darmstadt, Germany) and 0.5 g of red dye (Condi Alimentar, Camarate, Portugal) were dissolved in 50 mL of phosphate-buffered saline at 50 °C, yielding a thermoreversible hydrogel. The gel was injected warm (low viscosity) with a 30G NaviTip from the coronal/middle thirds until backflow at the orifice and then allowed to set for 5 min at room temperature. The 30G needle was not advanced to WL in the unprepared #10 canal; filling relied on capillarity and gentle pressure. To minimize air entrapment, the model was gently tapped/vibrated after injection to release microbubbles, and uniform coloring of both mesial canals (including the isthmus) was visually confirmed. This NaOCl-labile gel allowed the outcome to reflect both mechanical wash-out by irrigation and chemical degradation by NaOCl under a standardized delivery protocol.

### 2.3. Chemomechanical Procedures

A single experienced operator performed root canal preparation under an operative microscope (Zeiss Omni Pico, Carl Zeiss, Berlin, Germany) to ensure uniformity of the procedure. A total of 39 replicas were used, with 13 specimens randomly assigned to each of the three experimental groups using Research Randomizer (https://www.randomizer.org/). A gingival barrier was placed at the apical region of each model to simulate a closed-end canal system. Each replica was mounted and secured in a benchtop holder during all procedures to ensure stability.

All shaping procedures were performed using an X-Smart Plus motor (Dentsply Sirona, Ballaigues, Switzerland) and followed the manufacturer’s recommended protocols. Apical patency was confirmed with a size 10 K-file (Dentsply Tulsa Dental, Charlotte, NC, USA) inserted 0.5 mm beyond the WL. The WL was established 1 mm short of the apical foramen and confirmed visually. Each rotary instrument was used to prepare two canals and then discarded.

The operator delivered irrigation with 4% NaOCl through a 30-gauge IrriFlex^®^ needle (double side-vented, closed-end, flexible polyethylene; Produits Dentaires SA, Vevey, Switzerland), pre-curved and positioned 2 mm short of WL. Irrigation was administered intermittently between instruments at a consistent hand-delivery rate (~0.30 mL/s) established in pilot trials.

In the ProTaper Gold group (Dentsply Sirona, Ballaigues, Switzerland), instrumentation was performed in the sequence SX (19/.04), S1 (18/.02), S2 (20/.04), F1 (20/.07), and F2 (25/.08) at 300 rpm. Torque was 3.1 N·cm for SX, S1, F1, and F2 and 1.5 N·cm for S2. Irrigation with 4% NaOCl included a baseline rinse of 2 mL before SX, 9 mL distributed between instruments (2 mL after SX, 2 mL after S1, 2 mL after S2, and 3 mL after F1), and a final rinse of 3 mL.

The TruNatomy group (Dentsply Sirona, Ballaigues, Switzerland) was prepared using the Orifice Modifier (20/.08), followed by the Glider (17/.02) and then the Small (20/.04) and Prime (26/.04) shaping files, all at 500 rpm and 1.5 N·cm torque. Irrigation with 4% NaOCl comprised a baseline rinse of 2 mL before the Orifice Modifier, 9 mL distributed between instruments (2 mL after the Orifice Modifier, 2 mL after the Glider, 2 mL after the Small, and 3 mL after the Prime), and a final rinse of 3 mL.

Finally, in the SlimShaper group (Zarc4Endo, Gijón, Asturias, Spain), the sequence included ZS1 (15/.02–06, gold alloy), ZS2 (20/.04–03, pink alloy), and ZS3 (25/.04–03, blue alloy), all at 500 rpm and 3 N·cm torque. Irrigation with 4% NaOCl included a baseline rinse of 2 mL before ZS1, 9 mL distributed between instruments (3 mL after ZS1, 3 mL after ZS2, and 3 mL after ZS3), and a final rinse of 3 mL. The total volume of irrigant delivered per sample was standardized to 14 mL for all groups.

### 2.4. Image Acquisition and Quantification

Standardized photographs of each sample were acquired before and after canal instrumentation and irrigation using a high-resolution camera (Sony Alpha 7R IV, Sony Corporation, Tokyo, Japan; full-frame sensor 35.7 × 23.8 mm, 61 MP, 9504 × 6336 px) mounted on a fixed copy stand (Kaiser Repro Stand RS 2 XA, Kaiser Fototechnik GmbH & Co., KG, Buchen, Baden-Württemberg, Germany) to ensure identical positioning and lighting across images. A 90 mm macro lens (Sony FE 90 mm f/2.8 Macro G OSS, SEL90M28G, Sony Corporation, Tokyo, Japan) was used at 1:1 magnification (field of view ≈ 35.7 × 23.8 mm) with a working distance of ~140 mm (front element to specimen).

To maximize spatial resolution, images were captured at ISO 100, f/11 and saved as 14-bit RAW (lossless compressed); an electronic first-curtain shutter and Bluetooth remote (RMT-P1BT, Sony Corporation, Tokyo, Japan) were used to eliminate vibration. Illumination was provided by two 5500 K LED panels (Amaran P60x, Aputure Imaging Industries Co., Ltd., Shenzhen, China) placed at 45° with a neutral background; a calibrated scale (Stage micrometer, Edmund Optics Inc., Barrington, NJ, USA) was included in every frame. Each replica was stabilized vertically on an acrylic base to ensure consistent alignment across images.

Image processing and analysis were performed using ImageJ software (version 1.53, National Institutes of Health, Bethesda, MD, USA). Segmentation of the red-colored gel simulating the pulp tissue was performed using the “Apply Saved SIOX Segmentator” function under the Plugins > Segmentation menu. This tool allowed precise delineation of the gel-filled area within each canal.

The segmented areas were quantified in millimeters (mm^2^). The percentage of remaining tissue was calculated by comparing the stained area before and after treatment. The final results were expressed as the percentage of gel-like tissue remaining after instrumentation and irrigation.

### 2.5. Finite Element Analysis

A three-dimensional FEA was performed to evaluate stress distribution in the root structure following canal instrumentation with different NiTi systems. The initial STL model used for mesh generation corresponded to the non-instrumented tooth, obtained from a pre-operative micro-computed tomography (micro-CT) scan. Additional STL models representing post-instrumentation geometries were obtained from micro-CT scans of a natural tooth instrumented with each of the three systems and were used as a morphological reference. These models provided an accurate representation of dentin removal and final canal morphology and were independent from the 3D-printed replicas used for the experimental cleaning assessment.

Canal instrumentation was performed exclusively on the 3D-printed replicas, which were fabricated from a single master STL derived from the micro-CT scan of the natural tooth. This approach allowed all three instrumentation systems to be tested on identical anatomy, which would not have been possible using the original tooth. One representative replica per group was subsequently scanned using micro-CT to obtain the post-instrumentation geometries used for FEA and CFD analyses.

No intracanal filling material or coronal restoration was included in the finite element models. This simplification was intentionally adopted to isolate the effect of post-instrumentation canal geometry and dentin removal on stress distribution, without introducing confounding variables related to restorative materials, bonding interfaces, or restorative design.

All models were imported into ANSYS Workbench 2023 R1 (Ansys Inc., Canonsburg, PA, USA). Meshing was performed using tetrahedral elements. A mesh refinement and convergence analysis was conducted prior to the final simulations by progressively reducing the global element size and monitoring peak von Mises stress values. Mesh convergence was assumed when further refinement resulted in variations below 5%. Based on this analysis, a global element size of 0.4 mm was selected for all final models, resulting in approximately 39,556 nodes and between 22,872 and 30,126 elements, depending on the model.

Dentin was modeled as a homogeneous, isotropic, and linearly elastic material. A Young’s modulus of 18.6 GPa and a Poisson’s ratio of 0.31 were assigned, in accordance with values commonly reported for human dentin in the literature [14].

Boundary conditions were applied by constraining the apical third of the root with fixed support to simulate physiological anchorage. To reproduce clinical occlusal conditions, three loading scenarios were applied independently: (i) a vertical load of 225 N to represent average masticatory forces, (ii) a vertical load of 600 N to simulate maximum bite force, and (iii) an oblique load of 225 N applied at a 45° angle to mimic parafunctional or eccentric loading. In addition, a combined loading condition was simulated by simultaneously applying the 225 N vertical and 225 N oblique forces to approximate complex functional loading. Occlusal contacts were distributed over the central fossa and cusp tips in all scenarios [18,19,20].

All simulations were performed as static structural analyses. Von Mises stress was recorded as a comparative metric to evaluate stress concentration and distribution within the root structure under the different loading conditions. Although dentin exhibits brittle or quasi-brittle behavior and tensile stresses are more directly associated with fracture initiation, von Mises stress was selected to allow relative comparison between post-instrumentation geometries under identical boundary conditions, as commonly reported in endodontic FEA studies.

### 2.6. Computational Fluid Dynamics Simulation

CFD simulations were performed using ANSYS Fluent 2023 R1 (Ansys Inc., Canonsburg, PA, USA) to evaluate irrigant behavior in instrumented root canal systems. Three anatomically inspired CAD models were created based on micro-CT measurements of canal volume, curvature, and taper following preparation with each instrumentation system. Each model represented a mesial root of a mandibular molar with two canals connected by an isthmus and two independent apical foramina, consistent with the experimental setup. A customized three-dimensional model of the IrriFlex irrigation needle was positioned according to the final canal diameter and taper of each system.

The computational domain was discretized using unstructured tetrahedral elements, with local mesh refinement applied in the apical third and isthmus regions. A mesh refinement and independence analysis was performed by progressively increasing mesh density from approximately 200,000 to 720,000 elements. Final meshes containing approximately 690,000–720,000 elements resulted in less than 5% variation in peak apical velocity, wall shear stress, and dynamic pressure compared with the next finer mesh, ensuring numerical stability and grid convergence.

Simulations were conducted using a transient, pressure-based solver. The irrigant (sodium hypochlorite) was modeled as an incompressible Newtonian fluid with a density of 1.06 g·cm^−3^ and a dynamic viscosity of 0.001 Pa·s. A laminar flow model was adopted, consistent with previous experimental validation demonstrating that laminar simulations provide closer agreement with particle image velocimetry measurements than turbulence models under syringe irrigation conditions [21].

Gravity was applied along the longitudinal axis of the root (−9.8 m·s^−2^).

The irrigant inlet was defined at the lumen of the irrigation needle, where a constant mass flow rate of 0.00016 kg·s^−1^ was applied. Outlet boundary conditions were defined at the apical foramina and set to atmospheric pressure (101 kPa). All canal walls were modeled as rigid, no-slip boundaries.

Time-dependent simulations were performed over a total duration of 0.05 s using a time step of 1 × 10^−4^ s (500 steps). Second-order discretization schemes were applied to all governing equations. Convergence at each time step was defined as scaled residuals below 1 × 10^−4^, with monitored probes for velocity, wall shear stress, and pressure in the apical third and isthmus used to confirm solution stability. Simulation outputs included instantaneous and time-averaged velocity magnitude, wall shear stress, dynamic pressure, and particle tracking to characterize irrigant flow behavior within the canal system. Due to the computational complexity and the high degree of anatomical standardization, CFD simulations were performed on one representative post-instrumentation model per group and were intended to provide descriptive insight into irrigant flow behavior rather than statistically inferential comparisons.

### 2.7. Statistical Analysis

All statistical analyses were performed using IBM SPSS Statistics (version 29.0; IBM Corp., Armonk, NY, USA). The Shapiro–Wilk test was applied to evaluate the normality of the residual tissue data. As the data followed a normal distribution, a one-way analysis of variance (ANOVA) was conducted to compare the percentage of remaining gel-like tissue between the three instrumentation groups (SlimShaper, TruNatomy, and ProTaper Gold). Descriptive statistics were reported as mean ± standard deviation (SD).

Because of the limited number of computational models per group, the results of FEA and CFD simulations were only descriptive.

## 3. Results

### 3.1. Gel-like Pulp Tissue Remnant

Quantitative analysis confirmed that the initial amount of gel-like simulated pulp tissue was comparable in all samples (Table 1). No statistically significant differences were found between the groups (*p* > 0.05), indicating homogeneous filling before instrumentation.

**Figure 1:**
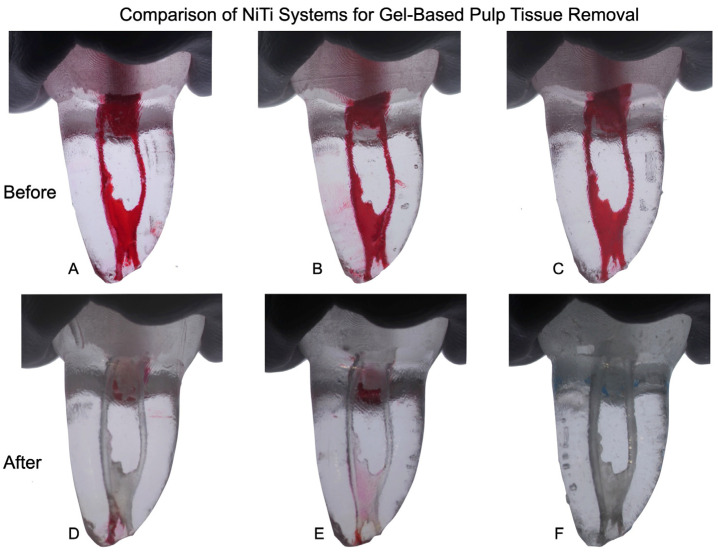
Representative images of gel-like pulp tissue before (**A**–**C**) and after (**D**–**F**) instrumentation with TruNatomy (**A**,**D**), SlimShaper (**B**,**E**), and ProTaper Gold (**C**,**F**). Post-instrumentation images illustrate variable amounts of residual tissue across systems.

This uniformity is visually supported by Figure 1, showing the pre-instrumentation condition of canals treated with TruNatomy (Figure 1A), SlimShaper (Figure 1B), and ProTaper Gold (Figure 1C). After treatment, the percentage of remaining tissue varied depending on the system. When evaluating the full canal length, ProTaper Gold showed the lowest average residual tissue (Figure 1F, 0.15 ± 0.25%), followed by SlimShaper (Figure 1E, 2.5 ± 3.81%) and TruNatomy (Figure 1D, 4.2 ± 5.12%), with no statistically significant differences (*p* > 0.05).

The apical third followed a similar trend, with mean values of 0.3 ± 0.6% for ProTaper Gold, 2.9 ± 4.1% for SlimShaper, and 8.9 ± 10.2% for TruNatomy. ANOVA analyses showed no statistically significant differences between the groups (*p* > 0.05).

In the middle third, the residual tissue was again lowest for ProTaper Gold (0.08 ± 0.4%), followed by TruNatomy (1.5 ± 2.1%) and SlimShaper (7.2 ± 9%). No statistically significant differences were found (*p* > 0.05).

Complete removal of the simulated tissue was observed in the coronal third in all groups (0%), as visually confirmed in Figure 1D–F.

A qualitative evaluation of the groups revealed that 3 of 13 ProTaper Gold samples (23.1%), 10 of 13 TruNatomy samples (76.9%), and 9 out of 13 SlimShaper samples (69.2%) had remnants of gel-like pulp material in at least one-third of the canal.

### 3.2. FEA Simulation Under Occlusal Loading

FEA in a single representative model for each group (uninstrumented, SlimShaper, TruNatomy, and ProTaper Gold) revealed qualitative differences in von Mises stress distribution under the simulated loading conditions. The peak stress values appeared higher after instrumentation, particularly under oblique and combined occlusal loads (Table 2).

Under vertical loading, the non-instrumented model showed the lowest, more localized stresses, mainly at the marginal ridges. In contrast, the instrumented models exhibited greater concentration in the pericervical region. Among the instrumented groups, ProTaper Gold presented the highest stresses, followed by TruNatomy, with SlimShaper showing the lowest, and a more centralized dispersion pattern for ProTaper Gold.

**Figure 2:**
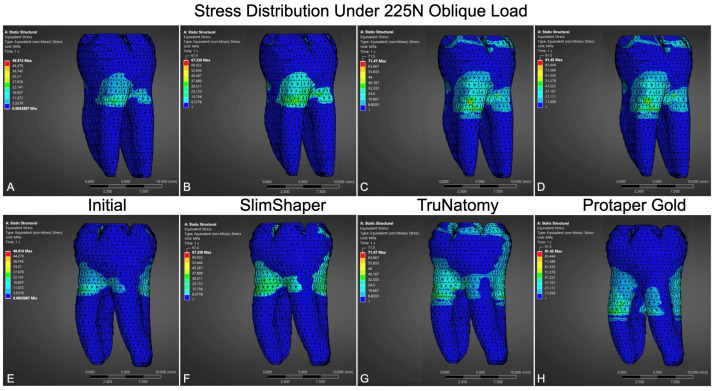
FEA showing von Mises stress distribution under a 225N oblique load at 45°. Frontal views are shown in images (**A**–**D**), and posterior views in images (**E**–**H**) for the uninstrumented model (**A**,**E**), SlimShaper (**B**,**F**), TruNatomy (**C**,**G**), and ProTaper Gold (**D**,**H**). All instrumented models exhibited increased stress concentrations, predominantly in the cervical and furcation regions.

With oblique loading (Figure 2), the same ranking was observed (ProTaper Gold > TruNatomy > SlimShaper > non-instrumented). Stress concentration was particularly evident in the coronal and cervical regions, including the furcation zone and pericervical dentin.

At maximum vertical load, all instrumented models exceeded the non-instrumented reference, with stress accumulation consistently observed in the pericervical dentin. ProTaper Gold again showed the highest values, TruNatomy showed intermediate values, and SlimShaper had the lowest values among the instrumented groups.

**Figure 3:**
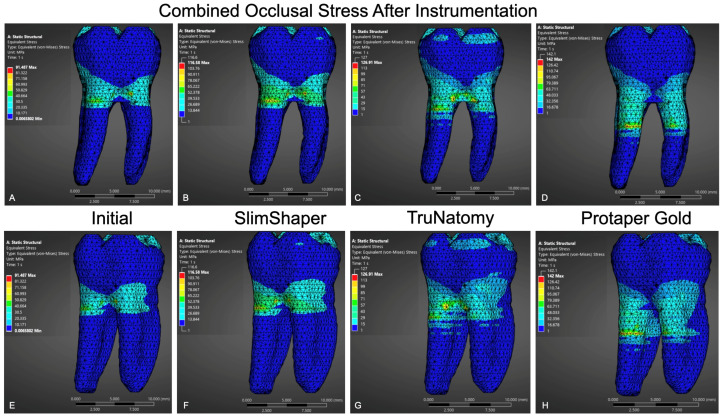
FEA showing von Mises stress distribution under combined occlusal loading: defined as the simultaneous application of a 225 N vertical load and a 225 N oblique load at 45°. Models shown are uninstrumented (**A**,**E**), SlimShaper (**B**,**F**), TruNatomy (**C**,**G**), and ProTaper Gold (**D**,**H**), with frontal and posterior views, respectively. Highest stress values were observed in the pericervical and furcation regions, particularly in the ProTaper Gold group.

Under combined loading (Figure 3), stresses increased in all models. ProTaper Gold exhibited the highest peaks, followed by TruNatomy and SlimShaper, while the non-instrumented model remained the lowest. The magnitude and spatial extent of stress distribution were broader in the instrumented models, with propagation toward the cervical and middle thirds of the roots.

### 3.3. Computational Fluid Dynamics Analysis

CFD analysis, performed on a single representative post-instrumentation model for each rotary system, revealed some differences in irrigant flow behavior in both the mesiobuccal (MB) and mesiolingual (ML) canals (Table 3). These results are presented descriptively based on representative models and should be interpreted as qualitative patterns of irrigant behavior rather than quantitative comparisons.

### 3.4. Particle Trajectories

**Figure 4:**
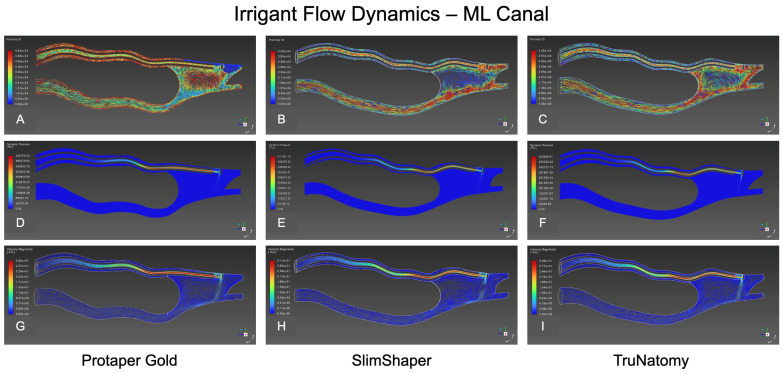
CFD visualization of irrigant behavior in the mesiolingual canal of representative post-instrumentation models. Results are shown for ProTaper Gold (**A**,**D**,**G**), SlimShaper (**B**,**E**,**H**), and TruNatomy (**C**,**F**,**I**). Top row (**A**–**C**): Particle ID maps illustrating the spatial distribution and trajectory of irrigant flow. Middle row (**D**–**F**): Dynamic pressure contours showing apical pressures and pressure gradients along the canal path. Bottom row (**G**–**I**): Particle motion fields depicting flow directionality and irrigant penetration into apical and lateral regions.

Mesiolingual Canal: In the single representative laminar models, all systems produced an apically directed core flow with a characteristic isthmus recirculation (Figure 4). ProTaper Gold generated a compact jet that hugged the outer wall toward the apical third, with a large clockwise vortex occupying the isthmus and visible particle convergence at its entrance (Figure 4A). SlimShaper showed earlier velocity decay and a smaller, more confined isthmus vortex (Figure 4B). TruNatomy was intermediate, with a narrower apical jet than ProTaper Gold and a well-defined isthmus vortex; particles entered and exited along the shear layer (Figure 4C).

**Figure 5:**
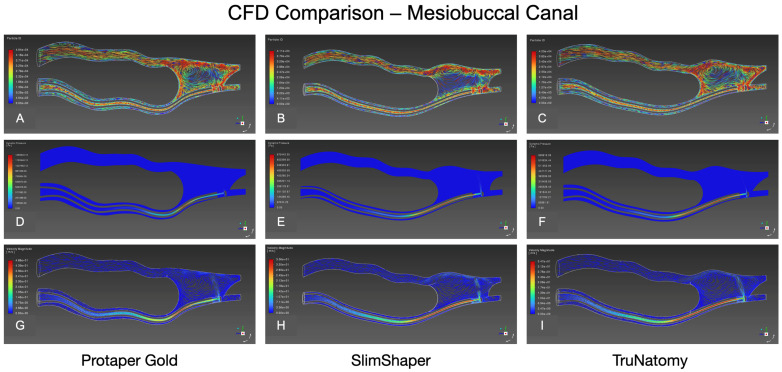
CFD visualization of irrigant dynamics in the mesiobuccal canal of representative post-instrumentation models for ProTaper Gold (**A**,**D**,**G**), SlimShaper (**B**,**E**,**H**), and TruNatomy (**C**,**F**,**I**). Particle ID maps (**A**–**C**) illustrate irrigant dispersion patterns, dynamic pressure contours (**D**–**F**) depict apical pressure distribution and pressure gradients, and time-averaged magnitude maps (**G**–**I**) show relative flow intensity along the canal trajectory. Results are intended to illustrate qualitative differences in irrigant behavior rather than quantitative comparisons.

Mesiobuccal Canal: Similar patterns were observed (Figure 5). ProTaper Gold displayed a coherent apical jet and pronounced isthmus recirculation with particle convergence at the cavity entrance (Figure 5A). SlimShaper exhibited a more subdued trajectory field and a smaller recirculation cell (Figure 5B). TruNatomy presented an intermediate jet with a persistent isthmus vortex (Figure 5C).

### 3.5. Dynamic Pressure

Mesiolingual Canal: Elevated dynamic pressure bands concentrated around the needle outlet and along the canal roof toward mid-root, attenuating apically in all models. Bands were visually higher for SlimShaper and TruNatomy than for ProTaper Gold (Figure 4D–F), suggesting greater resistance to fluid displacement in the more conservative tapers.

Mesiobuccal Canal: The same trend was found: localized high-pressure zones near the needle and along the roof, with SlimShaper and TruNatomy showing higher bands than ProTaper Gold (Figure 5D–F). Note: because color scales differ across systems, magnitude comparisons should rely on tabulated values rather than color alone.

### 3.6. Velocity Magnitude

**Figure 6:**
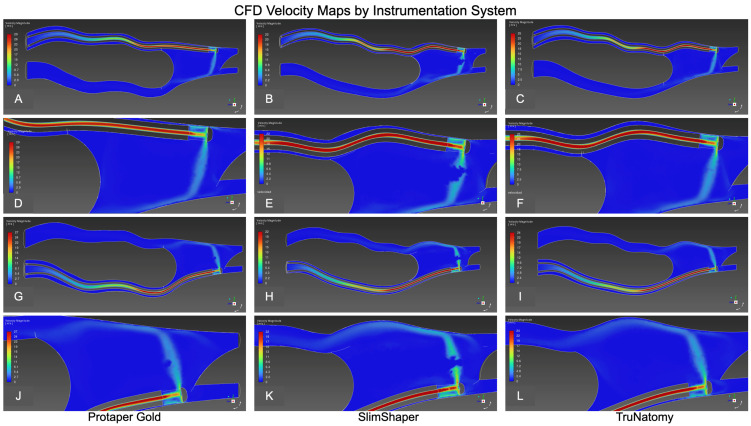
CFD-based time-averaged velocity magnitude maps in the mesiolingual and mesiobuccal canals of representative post-instrumentation models. Columns correspond to ProTaper Gold (**A**,**D**,**G**,**J**), SlimShaper (**B**,**E**,**H**,**K**), and TruNatomy (**C**,**F**,**I**,**L**). The top two rows (**A**–**F**) show time-averaged flow distribution in the mesiolingual canal, while the bottom two rows (**G**–**L**) show the mesiobuccal canal. The maps illustrate relative differences in flow continuity and apical penetration under identical irrigation conditions.

Mesiolingual Canal: All systems generated an apically directed high-velocity core. ProTaper Gold showed the broadest high-velocity footprint and deeper penetration of the fast core toward the apical third (Figure 4G and Figure 6A,D). SlimShaper presented a narrower core with earlier dissipation and larger low-velocity regions along the inner wall (Figure 4H and Figure 6B,E). TruNatomy behaved intermediately, maintaining a discernible apical jet with more limited lateral spread (Figure 4I and Figure 6C,F).

Mesiobuccal Canal: ProTaper Gold again exhibited the widest high-velocity bands and more extensive apical reach (Figure 5G and Figure 6G,J). SlimShaper showed a thinner, rapidly decaying core with widespread low-velocity zones (Figure 5H and Figure 6H,K). TruNatomy was intermediate, with a continuous apical jet but a smaller high-velocity region than ProTaper Gold (Figure 5I and Figure 6I,L).

### 3.7. Wall Shear Stress (WSS)

In the representative models, WSS concentrated primarily in the apical 1–2 mm and along the outer wall near the needle outlet, with additional hotspots at the isthmus mouth.

Mesiolingual Canal: ProTaper Gold showed localized high-WSS patches along the canal roof and near the isthmus entrance, with a peak of ≈18.7 Pa. SlimShaper displayed more continuous but lower-peak bands in this canal, peaking at ≈10.6 Pa. TruNatomy was intermediate, with discrete hotspots at curvature transitions and a peak of ≈8.6 Pa.

Mesiobuccal Canal: The distribution was comparable but of greater magnitude for ProTaper Gold, which exhibited the broadest high-WSS footprint over the outer wall and at the isthmus mouth, peaking at ≈18.8 Pa. SlimShaper showed elevated but more confined bands (≈10.4 Pa), whereas TruNatomy presented smaller, discontinuous high-WSS areas (≈8.8 Pa).

## 4. Discussion

The present study provides a comprehensive multimodal evaluation of three NiTi rotary systems—SlimShaper, TruNatomy, and ProTaper Gold—with experimental cleaning results, FEA, and CFD simulation.

None of the systems demonstrated statistically superior performance, confirming that under the standardized conditions used here, the taper profile alone did not determine significant differences in outcomes. Therefore, the null hypothesis was accepted. Even so, the experimental evaluation, FEA, and CFD provide a more nuanced understanding of how different shaping strategies interact with irrigation and dentin preservation.

The three NiTi systems evaluated in this study differ substantially in their shaping philosophy and taper design. ProTaper Gold is characterized by a progressively increasing taper and larger apical enlargement, which promotes greater canal widening, particularly in the coronal and middle thirds. In contrast, TruNatomy and SlimShaper are designed according to a more conservative shaping concept, with smaller, more uniform tapers intended to preserve radicular dentin, particularly in the pericervical region.

These design differences help contextualize the descriptive observations of the present study without implying performance superiority. The larger taper of ProTaper Gold was associated with higher irrigant velocities and wall shear stress, as well as increased stress concentrations in pericervical dentin, whereas the more conservative designs of TruNatomy and SlimShaper showed lower modeled stresses and higher dynamic pressure, reflecting greater resistance to irrigant flow. Despite these differences, cleaning efficacy did not differ significantly among systems, likely due to the standardized irrigation protocol and the use of a flexible needle positioned close to the WL.

Cleaning analysis showed that all systems left residual material, particularly in the apical third, which corroborates previous studies demonstrating that complete removal of tissue from mandibular molar mesial roots is rarely achieved [2,22]. This persistence of tissue is clinically relevant because remnants can serve as a substrate for bacterial colonization and survival, contributing to persistent apical periodontitis and long-term treatment failure [22]. Even small amounts of organic tissue may support biofilm growth, release inflammatory mediators, and maintain periapical infection, underscoring the importance of optimizing both shaping and irrigation to minimize remnants [2].

In this regard, while descriptive differences in residual gel were observed among the systems, statistical analysis showed no significant differences, reinforcing that anatomical complexities, particularly isthmuses and irregularities, plays a more decisive role than taper alone in determining cleanliness [2]. These findings mirror previous micro-CT and histobacteriologic studies that documented uninstrumented areas regardless of the shaping technique employed, highlighting that instrumentation alone cannot ensure sterility [2,3,22].

The apical third was consistently the most challenging region. Larger apical preparations have been traditionally advocated to improve irrigant penetration and apical cleaning, since increasing the apical diameter reduces vapor lock, enhances fluid exchange, and facilitates removal of debris [23,24,25]. CFD studies based on micro-CT-derived canal geometries have shown that canal enlargement, taper, and needle design significantly influence irrigant velocity, wall shear stress, and apical pressure, particularly in complex anatomies with isthmuses and apical ramifications [6,7,25,26,27]. The results of this study confirmed this tendency, as ProTaper Gold produced the highest irrigant velocities and shear stresses.

However, contrary to the assumption that this should translate into superior cleaning, no statistically significant differences were observed in the amount of gel removed between the larger-taper and smaller-taper shaping approaches [7].

This apparent divergence with some studies [28,29] can be explained by the systematic use of IrriFlex^®^ needles, which ensured insertion to 2 mm from the WL across all groups, compensating for the reduced lumen of the conservative systems [13]. Recent computational and experimental studies have shown that flexible, side-vented needles can effectively mitigate the hydraulic disadvantages of minimally shaped canals, supporting the interpretation that irrigation delivery design may be more decisive than taper alone in determining cleanliness [30,31,32,33]. From a clinical perspective, irrigation efficacy should be considered in relation to both canal shaping and the irrigation delivery method. Conventional metallic needles often require greater canal enlargement to allow deep penetration, whereas flexible, non-metallic needles can adapt to conservative preparations and facilitate irrigant delivery closer to the WL. Accordingly, adaptable irrigation systems may reduce the need for larger tapers, emphasizing a combined shaping–irrigation strategy rather than taper selection alone in clinical decision-making.

These patterns are consistent with previous micro-CT-based FEA studies showing that access cavity design and dentin preservation have a greater influence on stress distribution than canal taper alone, with conservative approaches reducing stress concentration in the pericervical dentin under occlusal loading [14,34,35]. In these simulations, a greater taper system (ProTaper Gold) exhibited higher descriptive peak stresses than more conservative designs. However, these stress distributions are derived from computational modeling and should not be interpreted as direct predictors of in vivo fracture risk.

These patterns are consistent with previous FEA studies associating larger tapers with increased modeled stress in structurally critical regions [36,37], whereas conservative shaping has been associated with lower modeled stresses and may mitigate stress concentration in pericervical dentin [34,38].

Clinically, endodontically treated teeth are restored with intracanal filling materials and coronal restorations, which may alter stress distribution and load transfer. The absence of restorative materials in the present models may therefore represent a worst-case structural scenario. Therefore, the FEA results should be regarded as illustrative of relative stress patterns under controlled conditions rather than as a direct extrapolation to clinical fracture outcomes.

However, FEA is a computational simulation based on assumed material properties and boundary conditions and does not measure fracture incidence or clinical longevity; therefore, these findings should be regarded as hypothesis-generating and interpreted with caution, particularly given the reliance on single representative models that may not fully reproduce clinical conditions.

Furthermore, it is important to distinguish between mechanical stresses generated during rotary instrumentation, which have been associated with dentinal microcrack formation, and the post-instrumentation biomechanical response of the root under functional occlusal loading. While several studies have investigated instrumentation-induced stresses of NiTi systems, FEA can also be applied to evaluate stress distribution in instrumented roots under functional loading conditions [39,40]. Accordingly, the present FEA should be interpreted as complementary to rather than overlapping with studies focusing on stresses generated during rotary instrumentation [41,42].

CFD analysis reinforced this interpretation. ProTaper Gold generated the highest irrigant velocities and wall shear stresses, reflecting enhanced apical fluid exchange, while SlimShaper exhibited the highest dynamic pressure, reflecting greater resistance to irrigant flow in narrower preparations. TruNatomy consistently showed intermediate patterns. These observations align with other CFD studies demonstrating that larger tapers facilitate flow and exchange but at the expense of greater dentin removal [7,8,43]. However, the present findings also indicate that, with optimized syringe delivery using a flexible, side-vented needle positioned close to the WL, conservative preparations can achieve cleanliness comparable to traditional tapers, challenging earlier assumptions that larger tapers inherently clean better [28]. This interpretation is consistent with one recent study that compared IrriFlex^®^ with conventional metallic needles across preparations of 30/.04 and 25/.06 and did not find significant differences when IrriFlex^®^ was used [44].

The clinical implication is that no single shaping philosophy can be universally recommended as superior [8]. Instead, shaping must be tailored to the tooth’s anatomy, balancing the need for apical enlargement to enhance irrigant delivery with the necessity of preserving radicular dentin to maintain fracture resistance [8]. Conservative approaches may safeguard long-term structural integrity but demand adjunctive irrigation strategies such as flexible needles or activation techniques to achieve effective cleaning [45]. Traditional larger tapers may simplify irrigant delivery but at the cost of higher stress accumulation in the pericervical dentin [46].

The limitations of this study should be acknowledged. Cleaning assessment was performed using a NaOCl-dissolvable hydrogel and 3D-printed canal replicas, which, although allowing high standardization and reproducibility, cannot fully replicate the biological complexity of pulp tissue and biofilms or the histological and mechanical properties of natural dentin. These simplifications may have influenced tissue removal mechanisms and irrigant–wall interactions compared with clinical conditions.

Another limitation is that only a single mandibular molar anatomy was investigated; therefore, the findings may not be directly extrapolated to teeth with different or more complex canal morphologies.

A primary limitation of the present study is that both FEA and CFD simulations were conducted on single representative models per group, precluding statistical validation. Consequently, the biomechanical and hydrodynamic results should be interpreted as descriptive and hypothesis-generating rather than predictive of clinical performance.

Additionally, the FEA did not model stresses generated during canal instrumentation, which have been associated with dentinal microcrack formation in previous studies, but focused instead on stress distribution under occlusal loading as influenced by post-instrumentation canal geometry. The assumption of isotropic and linearly elastic dentin also represents a simplification of its complex anisotropic behavior and should be considered when interpreting the FEA results.

## 5. Conclusions

Within the limitations of this study, no statistically significant differences were observed among the three NiTi systems in terms of cleaning efficacy, with all instruments leaving residual material, particularly in the apical third.

FEA and CFD provided descriptive data on stress distribution and irrigant behavior associated with different taper designs, without demonstrating system superiority. These findings indicate that taper design alone does not determine cleaning efficacy under standardized irrigation conditions.

## Figures and Tables

**Table 1:** Residual gel-like tissue in different regions of the root canal system. Values expressed as mean (standard deviation; range). Qualitative presence of tissue indicates samples with residual material in any third of the canal.

Measurement	ProTaper Gold	SlimShaper	TruNatomy
**All canal**			
Initial surface (mm^2^)	758.2 (29.1; 715–795)	755.5 (30.8; 700–790)	760.7 (28.4; 725–800)
Final surface (mm^2^)	1.12 (1.6; 0–5)	18.9 (29.5; 0–95)	23.1 (27.6; 0–88)
% Remaining tissue	0.15 (0.25; 0–0.8)	2.50 (3.81; 0–12.5)	4.20 (5.12; 0–16.5)
**Coronal third**			
Initial surface (mm^2^)	273.1 (25.8; 240–311)	276.4 (29.7; 230–320)	278.5 (27.5; 235–318)
Final surface (mm^2^)	0 (0; 0–0)	0 (0; 0–0)	0 (0; 0–0)
% Remaining tissue	0.00 (0.00; 0–0)	0.00 (0.00; 0–0)	0.00 (0.00; 0–0)
**Middle third**			
Initial surface (mm^2^)	252.9 (24.3; 210–288)	250.6 (26.1; 215–290)	253.3 (22.7; 218–286)
Final surface (mm^2^)	0.2 (0.4; 0–1.2)	18.1 (22.6; 0–78)	3.8 (5.4; 0–18.5)
% Remaining tissue	0.08 (0.4; 0–1.2)	7.20 (9; 0–28)	1.5 (2.1; 0–7)
**Apical third**			
Initial surface (mm^2^)	232.2 (21.5; 198–265)	228.5 (27.6; 184–267)	229.3 (24.1; 195–263)
Final surface (mm^2^)	0.7 (1.4; 0–4.2)	6.6 (9.8; 0–32)	20.4 (23.6; 0–78)
% Remaining tissue	0.3 (0.6; 0–1.8)	2.9 (4.1; 0–13.5)	8.9 (10.2; 0–35)
**Qualitative analysis**			
Samples with remnant tissue (%)	3/13 (23.1%)	9/13 (69.2%)	10/13 (76.9%)

**Table 2:** Peak von Mises stress (MPa) under vertical (225 N), oblique (225 N at 45°), maximum vertical (600 N), and combined loading. Values reported for instrumented and uninstrumented models.

Loading Condition	Uninstrumented	SlimShaper	TruNatomy	ProTaper Gold
Vertical load (225 N)	27.63	29.56	34.76	38.15
Oblique load (225 N at 45°)	49.81	67.34	71.47	91.45
Maximum vertical load (600 N)	45.12	49.8	52.1	61.5
Combined loading (All)	91.49	116.6	127.0	142.1

**Table 3:** Peak CFD values for time average (m/s), wall shear stress (Pa), and dynamic pressure (Pa) in Mesio Buccal (MB) and Mesio Lingual (ML) canals.

Parameter	Protaper Gold	SlimShaper	TruNatomy
Time Average (m/s)			
MB Canal	48.75	35.57	34.72
ML Canal	28.57	31.06	30.79
Wall shear stress (Pa)			
MB Canal	18.769	10.390	8.767
ML Canal	18.744	10.590	8.564
Dynamic pressure (Pa)			
MB Canal	1259.940	670.442	638.816
ML Canal	432.751	511.361	502.659

## Data Availability

The original contributions presented in this study are included in the article. Further inquiries can be directed to the corresponding authors.
